# Chronic inducible urticaria – having more than one is common and clinically relevant

**DOI:** 10.3389/fimmu.2025.1584771

**Published:** 2025-06-30

**Authors:** Marina Lebedkina, Elena Kovalkova, Gerelma Andrenova, Alexander Dushkin, Anton Chernov, Ekaterina Nikitina, Alexander Karaulov, Maryana Lysenko, Marcus Maurer, Emek Kocatürk, Daria Fomina

**Affiliations:** ^1^ Global Allergy and Asthma European Network (GA2LEN) Urticaria Center of Reference and Excellence (UCARE), Moscow City Research and Practical Center of Allergy and Immunology, City Clinical Hospital 52, Moscow Healthcare Department, Moscow, Russia; ^2^ Department of Clinical Immunology and Allergy, The First Sechenov State Medical University, Moscow, Russia; ^3^ Department of Surgery, Central Military Clinical Hospital Named After A.A. Vishnevsky, Moscow, Russia; ^4^ Life Improvement by Future Technologies (LIFT) Center, Skolkovo, Russia; ^5^ Therapy Department, The Russian National Research Medical University Named After N.I. Pirogov, Moscow, Russia; ^6^ Global Allergy and Asthma European Network (GA2LEN) Urticaria Center of Reference and Excellence (UCARE), Institute of Allergology, Charité – Universitätsmedizin Berlin, Berlin, Germany; ^7^ Department of Allergology and Immunology, Fraunhofer Institute for Translational Medicine and Pharmacology (ITMP), Berlin, Germany; ^8^ Department of Dermatology, Bahçeşehir University School of Medicine, Istanbul, Türkiye; ^9^ Department of Pulmonology, Astana Medical University, Astana, Kazakhstan

**Keywords:** urticaria, chronic inducible urticaria, wheal, angioedema, cold urticaria, symptomatic dermographism, cholinergic urticaria

## Abstract

**Background:**

Chronic inducible urticaria (CIndU) is characterized by wheals and/or angioedema (AE) for 6 weeks or more in response to specific and definite triggers. Individual patients can have more than one type of CIndU. Reports on this come from single cases or small case series, most epidemiological studies did not assess whether these urticaria are standalone or mixed.

**Objective:**

Determine the features of mixed CIndU and how they differ from standalone forms.

**Methods:**

In a prospective cohort study we performed provocative testing in 210 patients with CIndU. A total of 188 patients were included (125 with the standalone CIndU and 63 with the mixed CIndU. Within each group, patients were divided into subgroups: symptomatic dermographism (SD), cold (ColdU) and cholinergic urticaria (CholU).

**Results:**

Mixed CIndU most commonly were SD+ColdU (n=19, 30.2%), SD+CholU (n=15, 23.8%), SD+DPU (n=12, 19%) and ColdU+CholU (n=9, 14.3%). Comorbid chronic spontaneous urticaria (CSU) (50.8% *vs* 20.8, p<0.001) and AE (36.5% *vs*. 20%, p=0.014) were more common in patients with mixed CIndUs. In patients with mixed CIndUs, their onset time is more closely linked to each other (n=43; 68.2%) as compared to that of comorbid CSU (12 of 32, 37.5%). MixedSD patients are younger, show earlier onset of disease and have higher rates of AE and lower rates of atopic dermatitis (AtD), as compared to patients with standalone SD. MixedColdU patients have higher rates of comorbid CSU and lower rates of AtD, higher levels of total IgE and eosinophils. MixedCholU patients are older, have a high rate of comorbid CSU, a lower incidence of allergic disease.

**Conclusion:**

This study shows for the first time that each combinations of mixed CIndU represent a distinct phenotype with its own features. These phenotypes require special attention of specialists.

## 1 Introduction

Chronic inducible urticaria (CIndU) is a heterogeneous group of diseases characterized by wheals, angioedema (AE), or both for 6 weeks or more that develop in response to specific triggers (1). CIndU is divided into 2 subgroups - those with triggers that are physical factors [cold (ColdU), heat (HeatU), solar (SolarU), vibratory angioedema, delayed pressure urticaria (DPU) and symptomatic dermographism (SD)] and those with other triggers [cholinergic (CholU), aquagenic (AquaU), contact] (2, 3).

In comparison with chronic spontaneous urticaria (CSU), CIndU is characterized by a longer disease duration and lower rates of spontaneous remission (4, 5). Moreover, patients with CIndU, as compared to CSU, exhibit earlier disease onset, higher rate of concomitant allergic diseases (AD), lower rates of autoimmune diseases (AI), and lower rates of AE (6–9). Female gender predominates in most types of CIndU with the exception of CholU (5, 10–12).

The prevalence of CIndU is estimated to be at least 0.5% in the general population and up to 25% of all cases of chronic urticaria (CU) are CIndU (3). The most common CIndUs are SD (50-78% of all CIndU), ColdU (26.1%-37%), and CholU (6-30%) (1, 13–17). According to the results of an epidemiological study in Moscow, these three urticaria are also the most common: 50% SD, 10% CholU and 9% ColdU (18). Individual patients can have more than one type of CIndU (19–21). Previous reports generally entail single cases or small case series of CIndU (22–32), and do not describe whether cases were standalone or mixed. When such assessments were made, they primarily focused on comorbid CSU, with Pereira et al. reporting a rate of 21.5% (12) and Ozdemir et al. finding only 5% (33).

Thus, many questions remain to be answered:

1. Do patients with standalone and mixed forms of the most common CIndU (SD, ColdU, CholU) differ from each other?
2. How does the onset of the different urticaria vary between the groups and what might this mean?
3. How does each standalone form of urticaria (standalone SD (sSD), standalone CholU (sCholU), standalone ColdU (sColdU)) differ from patients with the same urticaria but in combination (mixedSD, mixedColdU, mixedCholU)?

We performed this study to address these knowledge gaps by describing the features of mixed CIndU (mixedCIndU) and comparing them with those of standalone CIndU (sCIndU). Comparisons of mixedCIndU and sCIndU, including such large numbers of patients, have not been done before.

## 2 Materials and methods

### 2.1 Study design

A single-center prospective study was conducted at one of the GA^2^LEN Urticaria Centers of Reference and Excellence (GA^2^LEN UCARE) (34) from January 2023 to January 2024. The study was approved by Local Ethics Committee (№ 08/0823 dated 30.08.2023), and all patients provided informed consent.

**Figure 1:**
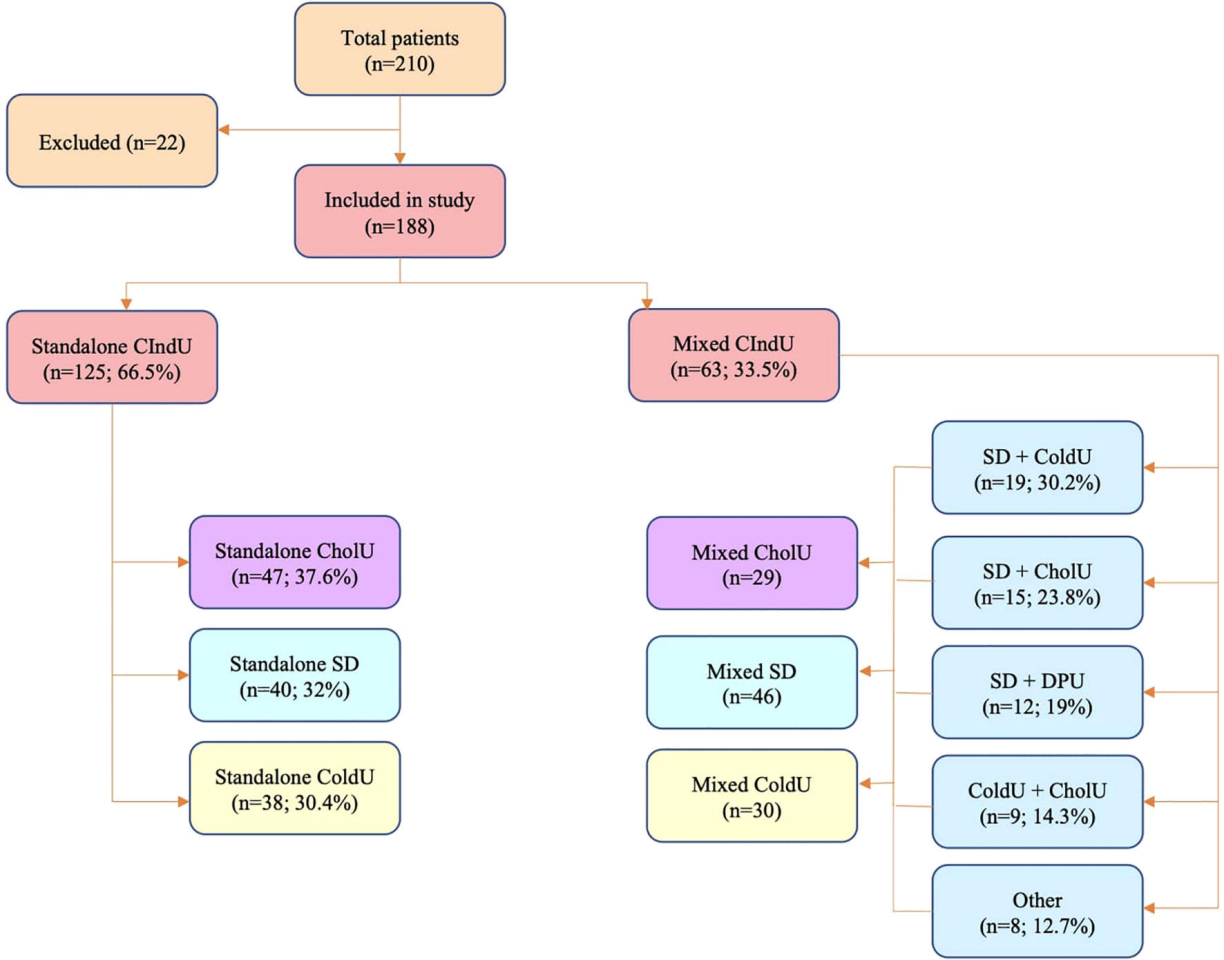
Study design. CIndU, chronic inducible urticaria; SD, symptomatic dermographism; CholU, cholinergic urticaria; ColdU, cold urticaria; DPU, Delayed Pressure Urticaria.

Inclusion criteria were as follows:

- older than 18 years of age;
- positive provocation test to 1 trigger in case of sCIndU and to 2 triggers in case of mixedCIndU;

Exclusion criteria were as follows:

- pregnancy, lactation (contraindications to provocation tests);
- the presence of more than two types of CIndU;
- patients with standalone or mixed forms not including patients with SD, CholU, Cold (as the most common forms);

During the follow-up period in the center, 210 patients with CIndU were identified by provocation testing. Of them 22 patients were excluded from the analysis according to the exclusion criteria (10 of them had 3 combined CIndU, 1 patient with 2 rare forms of CIndU (DPU+SolarU), 11 patients with another form of standalone CIndU (DPU, SolarU, HeatU, AquaU)). A total of 188 CIndU patients were included in the study (**Figure 1**). Of these, 63 patients had 2 types of CIndU and 125 patients had one. The following types of sCIndU were analyzed: sCholU (n=47), sSD (n=40) and sColdU (n=38). The following combinations in mixedCIndU were observed: SD+ColdU (n=19; 30.2%), SD+CholU (n=15; 23.8%), SD+DPU (n=12; 19%), ColdU+CholU (n=9; 14.3%), and other rarer combinations (n=8; 12.7%): SD+HeatU (n=1); ColdU+SolarU (n=2); CholU+DPU (n=2); CholU+HeatU (n=1); CholU+AquaU (n=2). All these combinations formed three main mixed groups: mixedSD (n=46), mixedColdU (n=30), mixedCholU (n=29).

The study compared patients with sCIndU and mixedCIndU. Patients were compared according to the following parameters: gender, age, duration of the disease, the age of onset, family history of any urticaria (CSU or CIndU), comorbid CSU, the presence of AE, systemic reactions, comorbid AD and AI and some laboratory parameters: total IgE, (0–130 IU/ml), C-reactive protein (CRP, 0–6 mg/L), eosinophil count (0–500 cells/mcL), basophil count (0–200 cells/mcL), presence of increased anti-thyroid peroxidase (TPO) IgG (anti-TPO-IgG).

On the next step the onset of different urticaria was analyzed:

1. Evaluation of CSU, CIndU, CIndU+CSU onset in patients with comorbid CSU in the mixedCIndU, mixedSD, mixedCholU, mixedColdU groups.
2. Evaluation of CSU, CIndU, CIndU+CSU onset in sCIndU, sSD, sCholU, sColdU groups.
3. In the mixedCIndU, mixedSD, mixedColdU, mixedCholU groups it was evaluated whether the onset of 2 CIndUs was simultaneous. In case of non-simultaneous onset, which urticaria started first.

Comparative analysis on the previously listed parameters was also performed between the following groups: sSD and mixedSD, sColdU and mixedColdU, sCholU and mixedCholU.

The study provides a descriptive characteristics of mixedCIndU. SCindU were compared between patients with the same type of urticaria from all mixedCIndU groups (sSD *vs* mixedSD, sColdU *vs* mixedColdU, sCholU *vs* mixedCholU).

The treatment schemes were not evaluated in this study because patients were included at different stages of treatment and dynamic response was not assessed. The requested washout period from the medications before the provocation was followed.

### 2.2 Statistical methods

Statistical analysis was performed using Python3.12 and statistical packages “scipy”. Quantitative features were assessed for normality using Shapiro-Wilk test. Quantitative features were described using mean (M), standard deviation (SD) and 95% confidence interval (CI95) with normal distribution or median (Me) with interquartile interval (Q1-3) with different distribution from normal. Categorical features were described using absolute value (n) and relative frequencies (%). The comparison of quantitative variables with normal distribution using Student’s t-test and Mann-Whiney U-test for non-normal distribution between two groups. The comparison between three or more groups regarding quantitative variables with normal distribution was performed using one-way analysis of variance and *post-hoc* comparisons was conducted using the Games-Howell test. For non-normal distribution comparison was conducted with Kruskal-Wallis test and Dunn’s test with Holm correction as a *post-hoc* method. Frequencies of categorical features were compared using Pearson chi-squared test. Differences were considered statistically significant at p<0.05.

## 3 Results

### 3.1 In patients with SD, CholU, or ColdU combination with another CIndU is common

Of 210 patients with CIndU, 188 patients had SD, CholU, or ColdU. Two-thirds (n=125, 66.5%) of these patients had them standalone, whereas one third (n= 63, 33.5%) in combination with another CIndU (**Figure 1**).

Of the 125 patients with sCIndU, 40 had SD (32%), 47 had CholU (37.6%), and 38 had ColdU (30.4%). Among patients with sSD, the prevalence of comorbid CSU was 40%, in patients with sColdU 2,6% and in patients with sCholU 19,1%. Of the 63 patients with mixedCIndU, SD+ColdU was the most common combination (30.2%), followed by SD+CholU (23.8%), SD+DPU (19%), ColdU+CholU (14.3%) and other rarer combinations (12.7%). Among patients with mixedSD, the prevalence of comorbid CSU was 54.3%, in mixedColdU 42.9%, in mixedCholU 48.3%. The prevalence of CSU in combinations was 42.1% in SD+ColdU, in SD+CholU 53.3%, in ColdU+CholU 44.4%, in SD+DPU 75.0%.

Of 86, 68, and 76 patients with SD, ColdU, and CholU, respectively, 53%, 44%, and 38% had their CIndU in combination with another CIndU.

### 3.2 MixedCIndUs was associated with CSU and AE

Patients with sCIndU did not differ from patients with mixedCIndU in gender distribution, age, age at onset of disease, disease duration, rates of systemic reactions, AI, or a family history of urticaria (**Table 1**). Comorbid CSU was more common in mixedCIndU patients compared to standalone (50.8% *vs* 20.8, p<0.001), as was AE (36.5% *vs*. 20%, p=0.014). As seen in the analysis of comorbid CSU described in the previous paragraph, in the mixedCIndU groups as well as in each of the combinations, its prevalence is 42.9-75%, whereas in the standalone groups only in the sSD group CSU occurs in 40% of cases and is almost absent in the sColdU group. Сomorbid atopic dermatitis (AtD) was more frequent in patients with sCIndU *vs* mixedCIndU, (14.4% *vs*. 0% (p=0.002)).

**Table 1:** Comparative analysis of patients with mixed and standalone CIndU.

Features	CIndU		*P* value
	Standalone (n=125)	Mixed (n=63)	
Gender: Female	66 (52.8%)	39 (61.9%)	0.235
Age, years	35 [24 – 48]	32 [25- 41.5]	0.218
Age of onset, years	22 [18 – 39]	25.5 [17 – 36.75]	0.903
Duration, month	48.00 [15.75 – 108]	42 [16.5 – 108]	0.966
Age of onset**, years	25,67 [17.46 - 34]	22 [18 - 39]	0.842
Duration**, month	48 [18 – 121.5]	48 [15.75 - 108]	0.822
Rate of comorbid CSU	26 (20.8%)	32 (50.8%)	**< 0.001**
AE	25 (20%)	23 (36.5%)	**0.014**
AE presence due to:			0.308
CSU	6 (42.9)%	8 (57.1)%
ColdU	13 (56.5%)	10 (43.5)%
CholU	6 (66.7%)	3 (33.3%)
DPU	0 (0%)	2 (100.0%)
Systemic reactions	14 (11.2%)	6 (9.7%)	0.751
AD:	53 (42.4%)	18 (28.6%)	0.065
ARC	47 (37.6%)	18 (28.6%)	0.219
BA	7 (56%)	1 (1.6%)	0.271
AtD	18 (14.4%)	0 (0%)	**0.002**
AI:	13 (10.4%)	8 (12.7%)	0.637
Family history of urticaria	7 (5,6%)	3 (4,8%)	1.000
Total IgE, IU/ml	112 [56 – 273]	140 [50 – 255]	0.942
CRP, mg/L	2.00 [0.12 – 4.42]	0,49 [0.09 – 1.95]	0.178
Eosinophil count, cells/mcL	160.00 [90 – 225]	200 [125 – 320]	**0.026**
Basophil count, cells/mcL	40.00 [20 – 50]	30.00 [5.04 – 50]	0.129
Increased anti-TPO-IgG	11 (17.7%)	5 (11.6%)	0.425

** Refers to any first onset urticaria (CSU and/or CIndU).

** Refers to the first onset CIndU.

CIndU, Chronic Inducible Urticaria; CSU, Chronic Spontaneous Urticaria; ColdU, Cold Urticaria; CholU, Cholinergic Urticaria; DPU, Delayed Pressure Urticaria; ARC, Allergic Rhinoconjunctivitis; BA, Bronchial Asthma; AtD, Atopic Dermatitis; AI, Autoimmune disease; AD, allergic diseases; AE, angioedema; CRP, C-reactive protein; anti-TPO-IgG, anti-thyroid peroxidase (TPO) IgG.

The bold values are statistically significant.

Laboratory parameters were similar, except for eosinophil counts, which were higher in patients with mixedCIndU *vs* sCIndU (p=0.026).

### 3.3 In patients with mixedCIndUs, their onset time is more close to each other as compared to that of comorbid CSU

**Figure 2:**
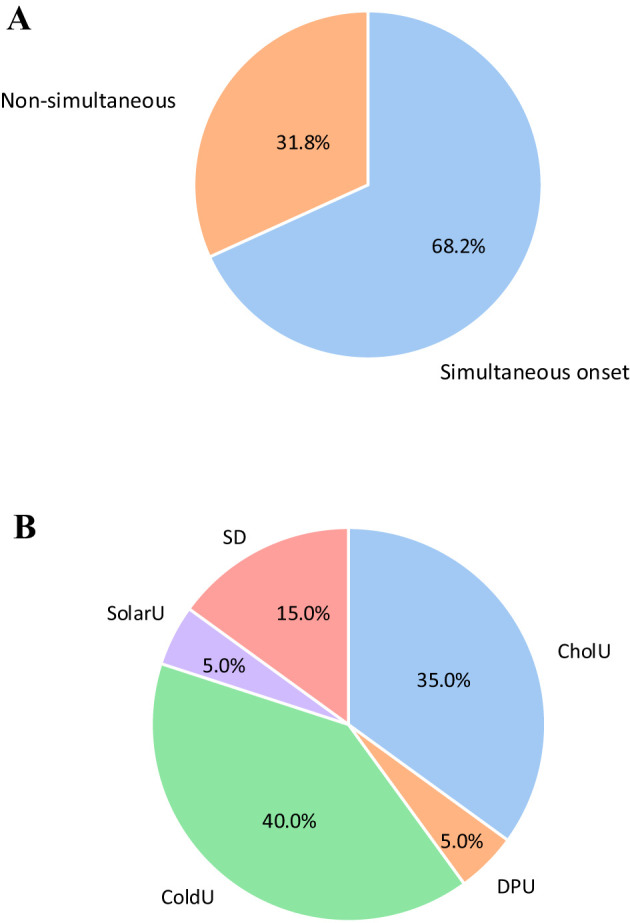
Assessment of the onset of CIndU in mixedCIndU group (n=63). **(A)** among all patient in mixed CIndU (n=63) 68.2% had simultaneous onset of 2 urticaria, 31.8% non-simultaneous onset. **(B)** with non-simultaneous onset, ColdU was the first in 40.0% of patients, CholU in 35.0%, SD in 15%, DPU in 5%, and SolarU in 5%. *SD, Symptomatic Dermographism; CholU, Cholinergic Urticaria; ColdU, Cold Urticaria; DPU, Delayed Pressure Urticaria; SolarU, Solar Urticaria*.

In two thirds (n=43; 68.2%) of 63 patients with mixedCIndU, both CIndUs started at the same time, i.e. within three months of each other (**Figure 2A**). This was similar for all combinations of mixedCIndU, where one was SD (67.4%), ColdU (60%), or CholU (62%) (**Table 2**). In non-simultaneous onset, ColdU was the first in 40.0% of patients, CholU in 35.0%, SD in 15%, DPU in 5%, and SolarU in 5% (**Figure 2B**).

**Table 2:** Comparison of 2 CIndU onset in mixedCIndU groups.

Features		mixedSD (n=46)	mixedColdU (n=30)	mixedCholU (n=29)	P
Simultaneous onset of two CIndUs		31 (67.4%)	18 (60%)	18 (62%)	0.786
First urticaria in non-simultaneous onset	CholU	4 (26.7%)	3 (25.0%)	7 (63.6%)	0.111
	ColdU	6 (40.0%)	8 (66.7%)	2 (18.2%)	
	DPU	1 (6.7%)	0 (0.0%)	0 (0.0%)	
	SD	4 (26.7%)	0 (0.0%)	2 (18.2%)	
	SolarU	0 (0.0%)	1 (8.3%)	0 (0.0%)	

CIndU, Chronic Inducible Urticaria; SD, Symptomatic Dermographism; CholU, Cholinergic Urticaria; ColdU, Cold Urticaria; DPU, Delayed Pressure Urticaria; SolarU, Solar Urticaria.

**Figure 3:**
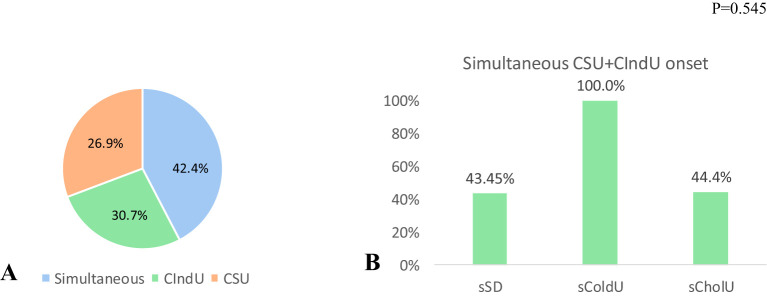
Assessment of the first presented urticaria in sCIndU among patients with CSU (n=26). **(A)** among all patient with comorbid CSU in sCIndU group 42.4% has simultaneous onset of CSU plus CIndU, 30.7% has CSU as first urticaria and 26.9% has CIndU as first urticaria. **(B)** Evaluation of the onset of simultaneous CSU plus CIndU in each of the standalone groups with comorbid CSU (sSD (n=7), sColdU (n=1), sCholU (n=9)). In sSD and sCholU 43.45% and 44.4% of patients had simultaneous debut of CSU plus CIndU, in sColdU simultaneous debut occurred in 100% (only 1 patient in a group), p=0.545. *CIndU, Chronic Inducible Urticaria; CSU, Chronic Spontaneous Urticaria; sSD, standalone Symptomatic Dermographism; CholU, standalone Cholinergic Urticaria; ColdU, standalone Cold Urticaria*.

**Figure 4:**
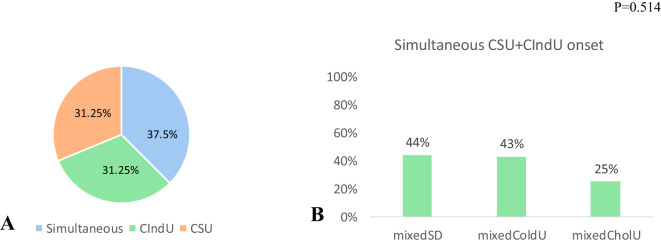
Assessment of the first presented urticaria in mixedCIndU among patients with CSU (n=32). **(A)** among all patient with comorbid CSU 37.5% has simultaneous onset of CSU+CIndU, 31.2% has CSU as first urticaria and 31.2% has CIndU as first urticaria. **(B)** In mixedSD (n=25) and mixedColdU (n=12) 44% and 43% of patients had simultaneous debut of CSU+CIndU, in mixedCholU (n=14) simultaneous debut occurred in 25%, p=0.514. *CIndU, Chronic Inducible Urticaria; CSU, Chronic Spontaneous Urticaria; SD, Symptomatic Dermographism; CholU, Cholinergic Urticaria; ColdU, Cold Urticaria*.

In contrast, comorbid CSU started at the same time with CIndU in less than half of patients in both groups, sCIndU (11 of 26, 42.4%, **Figure 3**) and mixedCIndU (12 of 32, 37.5%, **Figure 4**).

First urticaria in non-simultaneous onset was also assessed in each group (CholU, ColdU, DPU, SD, SolarU), with no significant differences between groups (p=0.111) (**Table 2**).

### 3.4 Patients with mixedSD are younger, show earlier onset of disease and have higher rates of angioedema and lower rates of AtD, as compared to patients with sSD

When sSD patients (n=40) were compared with mixedSD patients (n=46) (**Table 3**), both groups were predominantly female (72.5% *vs*. 60.9%, respectively, p=0.255), while patients with mixedSD were younger (30.5 [25.00; 35.75] *vs*. 40.00 [29.00; 50.00] years, respectively, p=0.002) with onset at an earlier age (27.34±13.30) *vs*. 34.15±12.46) years, respectively, p=0.017) and had a significantly higher rate of AE (37% *vs*. 10%, p=0.004) regardless the comorbid forms of CIndU and/or CSU. The occurrence of comorbid CSU did not differ between groups (54.3% for mixedSD *vs*. 40.0% for sSD, p=0.184), while the prevalence of AtD was significantly higher in the sSD group (12.5% *vs*. 0%, p=0.019), however the prevalence of AD did not differ between groups (25.5% for mixedSD *vs*. 35% for sSD, p=0.336).

**Table 3:** Comparative analysis of mixedSD *vs*. sSD.

Features	SD (n=86)		*P* value
	Standalone (n=40)	Mixed (n=46)	
Age, years	40.00 [29.00; 50.00]	30.50 [25.00; 35.75]	**0.002**
Gender: Female	29 (72.5%)	28 (60.9%)	0.255
Age of onset, years	34.15 (12.46)	27.34 (13.30)	**0.017**
Duration, months	25.00 [7.00; 108.00]	36.00 [13.25; 69.00]	0.330
Rate of comorbid CSU	16 (40.0%)	25 (54.3%)	0.184
AE	4 (10.0%)	17 (37.0%)	**0.004**
AE presence due to:			0.213
CSU	4 (36.4%)	7 (63.6%)	
CholU	0 (0.0%)	1 (100.0%)	
ColdU	0 (0.0%)	7 (100.0%)	
DPU	0 (0.0%)	2 (100.0%)	
Systemic reactions	0 (0.0%)	2 (4.4%)	0.496
AD:	14 (35%)	12 (25.5%)	0.336
ARC	12 (30.0%)	12 (26.1%)	0.687
BA	0 (0.0%)	1 (2.2%)	1.000
AtD	5 (12.5%)	0 (0.0%)	**0.019**
AI	3 (7.5)	6 (12.8)	0.498
Family history of urticaria	3 (7.5%)	2 (4.3%)	0.660
Total IgE, IU/ml	142.90 [65.30; 276.50]	125.60 [48.00; 237.00]	0.416
Eosinophil count, cells/mcL	200.00 [100.00; 230.00]	195.00 [122.50; 352.50]	0.553
Basophil count, cells/mcL	40.00 [20.00; 60.00]	30.00 [12.50; 65.00]	0.606
Anti-TPO-IgG	7 (26.9%)	4 (13.3%)	0.313

SD, Symptomatic Dermographism; CSU, Chronic Spontaneous Urticaria; CholU, Cholinergic Urticaria; ColdU, Cold Urticaria; DPU, Delayed Pressure Urticaria; ARC, Allergic Rhinoconjunctivitis; BA, Bronchial Asthma; AtD, Atopic Dermatitis; AI, Autoimmune disease; AD, allergic diseases; AE, angioedema; anti-TPO-IgG, anti-thyroid peroxidase (TPO) IgG.

The bold values are statistically significant.

### 3.5 Patients with mixedColdU have higher rates of comorbid CSU and lower rates of AtD, higher levels of total IgE and eosinophils

Women predominated in both mixedColdU (n=30) and sColdU (n=38) groups (83.3% and 78.9%, respectively) (**Table 4**), whereas there was a trend for younger age (36.79±13.8 *vs*. 43.68±15.19) years, p=0.063) and earlier disease onset (29.34±16.17) *vs*. 36.84±16.78) years, p=0.073) and a significantly higher incidence of comorbid CSU (42.9% *vs*. 2.6%, p<0.001) in the mixedColdU group compared sColdU. The frequency of AE did not differ (46.7% in mixedColdU *vs*. 36.8% in sColdU, p=0.414). AtD was significantly more common in the sColdU group (15.8% *vs*. 0%, p=0.035), while the prevalence of AD did not differ between groups (34.2% for mixedColdU *vs*. 36.7% for sColdU, p=0.833). The mixedColdU group had higher levels of total IgE (160.00 [90; 325] *vs*. 64.30 [35.20; 85.50] IU/ml, respectively, p=0.02), higher number of eosinophils (220.00 [180; 300] *vs*. 100.00 [37.5; 200] cells/mcL, p=0.002), and though not statistically significant, had lower basophil levels (30.00 [10.04; 30] *vs*. 42.00 [10; 100] cells/mcL, p=0.054).

**Table 4:** Comparative analysis of mixedColdU *vs*. sColdU.

Features	ColdU (n=68)		*P* value
	Standalone (n=38)	Mixed (n=30)	
Age, years	43.68 (15.19)	36.79 (13.8)	0.063
Gender: Female	30 (78.9%)	25 (83.3%)	0.761
Age of onset, years	36.84 (16.78)	29.34 (16.17)	0.073
Duration, months	30.00 [12.75; 84]	54.00 [23.50; 125.25]	0.317
Rate of comorbid CSU	1 (2.6%)	12 (42.9%)	**< 0.001**
AE	14 (36.8%)	14 (46.7%)	0.414
AE presence due to:	2 (66.7%)	1 (33.3%)	0.256
CSU	2 (100.0%)	0 (0.0%)	
CholU ColdU	10 (43.5%)	13 (56.5%)	
Systemic reactions	4 (10.5%)	3 (10.7%)	1.000
AD:	11 (36.7%)	13 (34.2%)	0.833
ARC	12 (31.6%)	10 (35.7%)	0.725
BA	3 (7.9%)	1 (3.6%)	0.631
AtD	6 (15.8%)	0 (0.0%)	**0.035**
AI	6 (20%)	7 (18.4%)	1.000
Family history of urticaria	3 (7.9%)	2 (7.1%)	1.000
Total IgE, IU/ml	64.30 [35.20; 85.50]	160.00 [90; 325]	**0.020**
Eosinophil count, cells/mcL	100.00 [37.5; 200]	220.00 [180; 300]	**0.002**
Basophil count, cells/mcL	42.00 [10; 100]	30.00 [10.04; 30]	0.054

ColdU, Cold Urticaria; CSU, Chronic Spontaneous Urticaria; CholU, Cholinergic Urticaria; ARC, Allergic Rhinoconjunctivitis; BA, Bronchial Asthma; AtD, Atopic Dermatitis; AI, Autoimmune disease; AD, allergic diseases; AE, angioedema.

The bold values are statistically significant.

### 3.6 Patients with mixedCholU are older, have a high rate of comorbid CSU, a lower incidence of AD

Patients with mixedCholU (n=29) were older than sCholU (n=47) patients (24.39±14.93) *vs*. 17.80±3.20) years, p=0.048) (**Table 5**) and were also more likely to have comorbid CSU (48.3% *vs*. 19.1%, p=0.007). However, the occurrence of AE did not differ between groups (33.3% for mixedCholU *vs*. 14.9% for sCholU, p=0.146). AD were significantly more common in the sCholU group (24.1% *vs*. 55.3%, p=0.008), i.e., allergic rhinoconjunctivitis (ARC) was more prevalent in the sCholU group (16.7% for mixedCholU *vs*. 48.9% for sCholU, p=0.008).

**Table 5:** Comparative analysis of mixedCholU *vs*. sCholU.

Features	CholU (n=76)		*P* value
	Standalone (n=47)	Mixed (n=29)	
Age, years	27 [22; 33]	26 [22; 38]	0.692
Gender: Male	40 (85.1%)	16 (66.7%)	0.122
Age of onset, years	17.80 (3.20)	24.39 (14.93)	**0.048**
Duration, months	60 [34.50; 115.50]	54.50 [36; 105]	0.616
Rate of comorbid CSU	9 (19.1%)	14 (48.3%)	**0.007**
Angioedema	7 (14.9%)	8 (33.3%)	0.146
AE presence due to:			0.056
CSU	1 (20.0%)	4 (80.0%)	
CholU	6 (75.0%)	2 (25.0%)	
ColdU	0 (0.0%)	2 (100.0%)	
Systemic reactions	10 (21.3%)	5 (21.7%)	0.965
Atopic diseases:	26 (55.3%)	7 (24.1%)	**0.008**
ARC	23 (48.9%)	4 (16.7%)	**0.008**
BA	4 (8.5%)	0 (0.0%)	0.292
AtD	7 (14.9%)	0 (0.0%)	0.087
AI comorbidity	3 (10.3%)	3 (6.4%)	0.669
Family history of urticaria	1 (2.2%)	2 (8.3%)	0.269
Total IgE, IU/ml	154,00 [66.80; 271]	144.50 [48.5; 291]	0.886
Eosinophil count, cells/mcL	150.00 [107.50; 222.50]	170.00 [100; 240]	0.483
Basophil count, cells/mcL	30.00 [27.50; 40.00]	30.00 [30.00; 40.00]	0.653
Anti-TPO-IgG	4 (11.1%)	2 (10.5%)	1.000

SD, Symptomatic Dermographism; CholU, Cholinergic Urticaria; ColdU, Cold Urticaria; CIndU, Chronic Inducible Urticaria; CSU, Chronic Spontaneous Urticaria; AI, Autoimmune disease; AD, allergic diseases; AE, angioedema.

The bold values are statistically significant.

## 4 Discussion

Here we present the largest study on the comparison of mixedCIndU and sCIndU. In our cohort, sCIndU was more common and accounted for two-thirds of all patients with CIndU. We found several differences between these groups. Comorbid CSU and AE were more common in patients with mixedCIndU. In mixedCIndU, their onset time was more closely linked to each other as compared to that of comorbid CSU. MixedCIndU differed from sCIndU in several features. Patients with mixedSD were younger, showed earlier onset of disease and had higher rates of AE and lower rates of AtD, as compared to patients with sSD. MixedColdU patients had higher rates of comorbid CSU and lower rates of AtD, higher levels of total IgE and eosinophils. MixedCholU patients were older, had a high rate of comorbid CSU, a lower incidence of AD.

The diagnosis of CIndU is particularly important, as the incidence of the disease continues to rise. For instance, findings from Moscow study (2017-2021) revealed a growing trend in all types of CIndU (18). In particular, the number of patients with CholU increased by more than three times, and ColdU by 2.5 times in 4 years. In our study, 66.5% of all patients had sCIndU and 33.5% had mixedCIndU. We observed that 53% of SD had mixedSD, 44% of ColdU had mixedColdU and 38% of CholU had mixedCholU. Despite the fact that sCIndU predominate, the presence of mixedCIndU in one third of patients may indicate a misdiagnosis of this disease worldwide. The most frequent combinations in our study were SD+ColdU (30.2%), SD+CholU (23.8%), SD+DPU (19%) and ColdU+CholU (14.3%). These data nearly match with our previous study (except for the SD+ColdU combination), where also the most frequent combinations were SD+CholU (13.9%), SD+DPU (25%) and ColdU+CholU (16.7%) (9).

In the mixedCIndU group, the female sex predominates (61.9%). In the sCIndU, the distribution of sex is almost equal (52.8% of women), but the difference between the groups is not significant. When analyzed further, it turned out that the prevalence of female was higher for sColdU, mixedColdU, sSD, mixedSD, but in both sCholU and mixedCholU male absolutely predominate (85.1% and 66.7%, respectively). Our findings align with findings from previous studies, where female sex predominance is typical for all CIndUs, where the prevalence of female reaches 74% (11), but for CholU male sex predominates (35, 36).

The onset age of CIndU varies between standalone and mixed forms, aligning with known peak ages of CIndU incidence (20s-30s) (2, 3). In our study sSD tends to manifest later, with mixedSD appearing significantly earlier (27 *vs*. 34 years respectively). Similarly, mixedColdU present at a younger age than sColdU (37 *vs*. 29 years respectively), though the difference is less statistically strong. In contrast, mixedCholU has a later onset compared to sCholU, with onset typically occurring in adolescence or young adulthood (24 *vs*. 18 years, respectively). Notably, earlier onset in some forms, like ColdU and CholU, may be associated with a longer disease duration (21, 37) underscoring the distinct patterns of onset and progression between mixed and standalone types.

Estimating duration in our cohort is challenging, as none of the patients were in remission, suggesting the true duration exceeds the nearly 4 years observed for both mixed and standalone forms. It is known that the duration of CIndU is often longer than CSU, with lower rates of remission at 1 year and some studies showing the lowest rate of remission after 10 years for ColdU (13). No reliable difference was obtained in any of the 3 forms of standalone and mixedCIndU, but this difference may appear when evaluated after the disease remission. It would be interesting to know which of the two mixedCIndU forms would go into remission and whether the groups would differ.

When the mixed and standalone groups were compared for the presence of AD and AI, there was a trend for a higher incidence of AD in the sCIndU group (42.4% *vs*. 28.6%, p=0.065). The groups differed significantly only in the prevalence of AtD (0% and 12.5% respectively, p=0.002). Interestingly, when we analyzed each of the three mixed and standalone urticaria forms, AtD was significantly more common in the sSD, sColdU, and sCholU groups and completely absent in the mixed groups. This finding suggests that the presence of two urticaria may have a protective effect against AtD, but the potential mechanisms underlying this phenomenon remain to be recognized. MixedCholU and sCholU differ in the prevalence of allergic diseases, which predominates in sCholU (24.1% *vs*. 55.3%, p=0.008), in particular the prevalence of ARC is significantly different (49% for sCholU and 17% for mixedCholU).

Regarding the laboratory parameters between groups, a higher level of eosinophils was observed in the mixed group (200 *vs*. 160 cells/mcL). The mixedColdU differs from sColdU in some laboratory parameters. The mixedCIndU group had significantly higher levels of total IgE (160 *vs*. 64.3 IU/ml). Moreover, in the mixedColdU total IgE is higher than the reference values, and the significance of these data remains to be determined. Another interesting difference is the difference in eosinophils, which are significantly higher in the mixedColdU group (220 *vs*. 100.00 cells/mcL). A trend towards lower levels of basophils is also noted. The role of these cells in the pathogenesis of ColdU is currently poorly understood.

CIndU frequently coexists with CSU, especially SD, which affects up to 35% of CSU patients (38). The presence of CIndU in CSU patients generally predicts a more severe, prolonged disease course with lower remission rates, as indicated by various studies (6, 39–41). Findings from this study also suggest that mixedCIndU, particularly combinations with ColdU and CholU, is associated with an increased risk of CSU, with higher CSU rates in mixedCIndU patients compared to sCIndU cases.

As for the prevalence of the presence of AE, it is significantly more frequent in the mixedCIndU group (36.5% *vs*. 20%). The same is observed in the SD group (37.0% *vs*. 10.0%). AE was also prevalent in mixedColdU and mixedCholU, but the difference between these groups was not statistically significant. Given the high prevalence of CSU in the mixed CIndU groups, it was important to understand the potential source of AE. Potential risk factors such as comorbid CSU and 1 of the CIndUs (CholU, ColdU, DPU) were analyzed. It appeared that there was no difference between groups in the causes of AE, which may suggest that it is the combination of two urticaria that influences AE development, especially in the SD group. Interestingly, in our previous study, patients with CSU+CIndU were less likely to have AE than patients with standalone CSU (53% *vs*. 72%) (9). This may be explained by the fact that the majority of patients in the previous study had a standalone type of CIndU (85.4%).

Another important aspect was the analysis of urticaria onset in all groups. It is noteworthy that in the mixedCIndU (n=63), more than a third of patients (37.5%) reported a simultaneous onset of CSU+CIndU, and almost all patients (10 of 12) had a simultaneous onset of all three urticaria. When comparing the 3 mixed groups with each other, in mixedSD and mixedColdU almost half of the patients (44% and 43% respectively) had simultaneous CSU+CIndU onset, and in mixedCholU a quarter (25%) of the patients had a simultaneous onset. In our previous study comorbid CIndUs most commonly started together with CSU, rather than before or after (9). In standalone groups, simultaneous onset of CSU+CIndU occurred in 42.4% of cases, while the sSD (43.45%), sColdU (n=1, 100%), and sCholU (44.4%) groups did not differ significantly. This high percentage of simultaneous onset may suggest a common pathogenetic mechanism involving autoimmunity and/or autoallergy. For example, it was previously reported that type I autoimmunity, also known as autoallergy, is believed to play an important role in the pathogenesis of not only CSU, but also CIndU (36).

When we examined the onset of each CIndU in mixedCIndU cases, 68.2% had a simultaneous onset - a proportion that was even higher than observed in CSU+CIndU combinations. This suggested that the pathogenic pathways of two coexisting CIndUs were more closely interconnected compared to those involving CSU. The pathogenesis of each CIndU remains unclear, but evidence suggests autoallergens play a role, such as sweat acting as an autoallergen in CholU. Studies have shown that CholU patients may experience a type I allergy to their own sweat (42, 43), potentially triggered by the fungal protein MGL_1304 from *Malassezia globosa* (44, 45). Although autoallergens in ColdU have not yet been identified, immunological mechanisms are likely involved (46–48), and Omalizumab’s effectiveness across CIndUs supports an IgE-dependent pathway (49–53).

However, whether there are common autoallergens for CIndU and CSU and for different types of CIndU is a subject of further investigation.

Although the efficacy of therapy was not evaluated in our study, according to the literature, the recommended therapy with non-sedating H1-antihistamines (nsH1-AH), even in escalated doses, can be ineffective in some patients with CIndU. Kocatürk et al. (35) showed with standard doses of nsH1-AH the response of patients with CIndU was lower compared to CSU (20.9% *vs*. 37.9%, respectively), and 79.1% of patients with CIndU did not achieve control (UCT≥12). In the study by Maurer et al (7), of 19.3% of patients with CIndU on standard doses of nsH1-AH, only 10.3% of them were able to continue the same dose of therapy after 2 years. This fact may suggest that although histamine is one of the major mediators released during mast cell activation, it is only one of a broad spectrum of proinflammatory mediators (54, 55). Thus, treatment targeting the mast cell as a major player in the pathogenesis of CIndU may be more effective than treatment of nsH1-AH targeting the mediator alone. For example, the use of anti-IgE therapy with omalizumab has been shown to be effective, including patients with mixedCIndU. For example, a study by Vieira et al. (32) showed a positive effect of omalizumab in a patient with a combination of CSU, DPU and SD. In the study by Nettis et al. (56), omalizumab was effective in a patient with CSU, ColdU, SD and CholU. Since experience of treatment with omalizumab in patients with mixedCIndU is limited to single clinical cases, further study is needed.

Our study has several strengths and limitations. As for the former, for the first time, the question of the incidence of urticaria in standalone and mixedCIndU groups was raised. and features of urticaria in mixed and standalone CIndU as well as in subgroups (sSD, sColdU, sCholU, mixedSD, mixedColdU, mixedCholU) were assessed. As for limitations, this study does not evaluate the efficacy of therapy. There was also no comparison of the level of thresholds in different groups. Future studies could aim in determining the dominant urticaria type in mixedCIndU cases, offering an opportunity to compare patient profiles and understand how this dominant type shapes the disease experience.

## 5 Conclusion

The results of the study showed that SD, CholU, ColdU are the most common urticaria, both in standalone and mixed forms. MixedCindU may be a risk factor for the development of comorbid CSU and AE. There may also be a protective effect of two forms of urticaria on the development of AtD. Each of the mixed and standalone CIndU groups differs in a number of features. The high prevalence of simultaneous onset of CSU+CIndU and two CIndUs may suggest a common pathogenesis, which requires further investigation to determine the specific mechanisms that trigger the other urticaria. This study shows for the first time that each combinations of mixedCIndU represent a distinct phenotype with its own features. These phenotypes require special attention of specialists. Data accumulation is required to understand not only the clinical picture and course of the disease, but also the response to therapy.

## Data Availability

The data that support the findings of this study are available from the corresponding author upon reasonable request.

## References

[1] Magerl M Altrichter S Borzova E Giménez-Arnau A Grattan CE Lawlor F. The definition, diagnostic testing, and management of chronic inducible urticaria5/23/25 9:57:00 PMs - The EAACI/GA(2) LEN/EDF/UNEV consensus recommendations 2016 update and revision. Allergy. (2016) 71:780–802. doi: 10.1111/all.12884 26991006

[2] Zuberbier T Abdul Latiff AH Abuzakouk M Aquilina S Asero R Baker D. The international EAACI/GA²LEN/EuroGuiDerm/APAAACI guideline for the definition, classification, diagnosis, and management of urticaria. Allergy. (2022) 77:734–66. doi: 10.1111/all.15090 34536239

[3] Abajian M Schoepke N Altrichter S Zuberbier T Maurer M. Physical urticarias and cholinergic urticaria. Immunol Allergy Clin North Am. (2014) 34:73–88. doi: 10.1016/j.iac.2013.09.010 24262690

[4] Jain SV Mullins RJ. Cold urticaria: a 20-year follow-up study. J Eur Acad Dermatol Venereol. (2016) 30:2066–71. doi: 10.1111/jdv.13841 27422852

[5] Kring Tannert L Stahl Skov P Bjerremann Jensen L Maurer M Bindslev-Jensen C. Cold urticaria patients exhibit normal skin levels of functional mast cells and histamine after tolerance induction. Dermatology. (2012) 224:101–5. doi: 10.1159/000336572 22398751

[6] Curto-Barredo L Archilla LR Vives GR Pujol RM Giménez-Arnau AM. Clinical features of chronic spontaneous urticaria that predict disease prognosis and refractoriness to standard treatment. Acta Derm Venereol. (2018) 98:641–7. doi: 10.2340/00015555-2941 29648675

[7] Maurer M Giménez-Arnau A Ensina LF Chu CY Jaumont X Tassinari P. Chronic urticaria treatment patterns and changes in quality of life: AWARE study 2-year results. World Allergy Organ J. (2020) 13:100460. doi: 10.1016/j.waojou.2020.100460 32983330 PMC7493083

[8] Rossi O Piccirillo A Iemoli E Patrizi A Stingeni L Calvieri S. Socio-economic burden and resource utilisation in Italian patients with chronic urticaria: 2-year data from the AWARE study. World Allergy Organ J. (2020) 13:100470. doi: 10.1016/j.waojou.2020.100470 33343800 PMC7726718

[9] Kovalkova E Fomina D Borzova E Maltseva N Chernov A Serdoteckova S. Comorbid inducible urticaria is linked to non-autoimmune chronic spontaneous urticaria: CURE insights. J Allergy Clin Immunol Pract. (2024) 12:482–90. doi: 10.1016/j.jaip.2023.11.029 38008357

[10] Rujitharanawong C Tuchinda P Chularojanamontri L Chanchaemsri N Kulthanan K. Cholinergic urticaria: clinical presentation and natural history in a tropical country. BioMed Res Int. (2020) 2020:7301652. doi: 10.1155/2020/7301652 32596363 PMC7273400

[11] Silpa-archa N Kulthanan K Pinkaew S. Physical urticaria: prevalence, type and natural course in a tropical country. J Eur Acad Dermatol Venereol. (2011) 25:1194–9. doi: 10.1111/j.1468-3083.2010.03951.x 21175877

[12] Pereira ARF Motta AA Kalil J Agondi RC. Chronic inducible urticaria: confirmation through challenge tests and response to treatment. Einstein (Sao Paulo). (2020) 18:eAO5175. doi: 10.31744/einstein_journal/2020ao5175 32667419 PMC7334002

[13] Maurer M Fluhr JW Khan DA. How to approach chronic inducible urticaria. J Allergy Clin Immunol Pract. (2018) 6:1119–30. doi: 10.1016/j.jaip.2018.03.007 30033913

[14] Dressler C Rosumeck S Werner RN Magerl M Metz M Maurer M. Executive summary of the methods report for ‘The EAACI/GA2 LEN/EDF/WAO Guideline for the Definition, Classification, Diagnosis and Management of Urticaria. The 2017 Revision and Update’. Allergy. (2018) 73:1145–6. doi: 10.1111/all.13414 29336489

[15] Sánchez-Borges M Ansotegui IJ Baiardini I Bernstein J Canonica GW Ebisawa M. The challenges of chronic urticaria part 2: Pharmacological treatment, chronic inducible urticaria, urticaria in special situations. World Allergy Organ J. (2021) 14:100546. doi: 10.1016/j.waojou.2021.100546 34141049 PMC8188551

[16] Prosty C Gabrielli S Le M Ensina LF Zhang X Netchiporouk E. Prevalence, management, and anaphylaxis risk of cold urticaria: A systematic review and meta-analysis. J Allergy Clin Immunol Pract. (2022) 10:586–96. doi: 10.1016/j.jaip.2021.10.012 34673287

[17] Kontou-Fili K Borici-Mazi R Kapp A Matjevic LJ Mitchel FB. Physical urticaria: classification and diagnostic guidelines. EAACI position paper Allergy. (1997) 52:504–13. doi: 10.1111/j.1398-9995.1997.tb02593.x 9201361

[18] Fomina DS Maltseva NP Serdotetskova SA Danilycheva IV Lebedkina MS Mikhaylova VI. Epidemiology of chronic inducible urticaria in Moscow. Russian J Allergy. (2022) 19:317–27. doi: 10.36691/RJA1573

[19] Mao M Yuan Y Xiao Y Peng C Chen X Li J. Clinical difference between single subtype and mixed subtype chronic urticaria: A retrospective study. Indian J Dermatol Venereol Leprol. (2022) 88:171–6. doi: 10.25259/IJDVL_257_20 34491667

[20] Neittaanmäki H. Cold urticaria. Clinical findings in 220 patients. J Am Acad Dermatol. (1985) 13:636–44. doi: 10.1016/s0190-9622(85)70208-3 4078052

[21] Deza G Brasileiro A Bertolín-Colilla M Curto-Barredo L Pujol RM Giménez-Arnau AM. Acquired cold urticaria: Clinical features, particular phenotypes, and disease course in a tertiary care center cohort. J Am Acad Dermatol. (2016) 75:918–24. doi: 10.1016/j.jaad.2016.06.017 27485164

[22] Torchia D Francalanci S Bellandi S Fabbri P. Multiple physical urticarias. Postgrad Med J. (2008) 84:e1–2. doi: 10.1136/pgmj.2007.062760 18230741

[23] Diehl KL Erickson C Calame A Cohen PR. A woman with solar urticaria and heat urticaria: A unique presentation of an individual with multiple physical urticarias. Cureus. (2021) 13:e16950. doi: 10.7759/cureus.16950 34513518 PMC8418825

[24] Cheon HW Han SJ Yeo SJ Lee SH Kim MJ Kim SH. A case of combined cholinergic and cold urticaria. Korean J Intern Med. (2012) 27:478–9. doi: 10.3904/kjim.2012.27.4.478 PMC3529252 23269894

[25] Zimmer S Peveling-Oberhag A Weber A Gilfert T Rady-Pizarro U Staubach P. Unique coexistence of cold and solar urticaria and its efficient treatment. Br J Dermatol. (2016) 174:1150–2. doi: 10.1111/bjd.14354 26678859

[26] Mathelier-Fusade P Aissaoui M Chabane MH Mounedji N Leynadier F. Association of cold urticaria and aquagenic urticaria. Allergy. (1997) 52:678–9. doi: 10.1111/j.1398-9995.1997.tb01055.x 9226069

[27] Ohashi T Kan Y Takahashi H Yoneta D Kase K Sumikawa Y. Cold urticaria comorbid with heat urticaria: A case report. J Dermatol. (2020) 47:e325–6. doi: 10.1111/1346-8138.15463 32578246

[28] Pitsios C Vithoulka A Roumana A Kompoti E Kontou-Fili K. Multiple physical urticarias: report of three cases and review of the literature. Allergy Asthma Proc. (2003) 24:313–7. 14619330

[29] Farnam J Grant JA Lett-Brown MA Lord RA Russell WL Henry DP. Combined cold- and heat-induced cholinergic urticaria. J Allergy Clin Immunol. (1986) 78:353–7. doi: 10.1016/s0091-6749(86)80089-6 3734288

[30] Sanz de Galdeano C Gardeazabal J Oleaga JM Diaz-Perez JL. Solar urticaria and cold urticaria in the same patient. Br J Dermatol. (1994) 131:143–5. doi: 10.1111/j.1365-2133.1994.tb08479.x 8043414

[31] Oda Y Fukunaga A Tsujimoto M Hatakeyama M Washio K Nishigori C. Combined cholinergic urticaria and cold-induced cholinergic urticaria with acquired idiopathic generalized anhidrosis. Allergol Int. (2015) 64:214–5. doi: 10.1016/j.alit.2014.12.004 25838106

[32] Vieira Dos Santos R Locks Bidese B Rabello de Souza J Maurer M. Effects of omalizumab in a patient with three types of chronic urticaria. Br J Dermatol. (2014) 170:469–71. doi: 10.1111/bjd.12628 24102388

[33] Ornek Ozdemir S Kuteyla Can P Degirmentepe EN Cure K Singer R Kocaturk E. A comparative analysis of chronic inducible urticaria in 423 patients: Clinical and laboratory features and comorbid conditions. J Eur Acad Dermatol Venereol. (2024) 38(3):513–20. doi: 10.1111/jdv.19637 37991240

[34] Maurer M Metz M Bindslev-Jensen C Bousquet J Canonica GW Church MK. Definition, aims, and implementation of GA(2) LEN Urticaria Centers of Reference and Excellence. Allergy. (2016) 71:1210–8. doi: 10.1111/all.12901 27038243

[35] Kocatürk E Can PK Akbas PE Copur M Degirmentepe EN Kızıltac K. Management of chronic inducible urticaria according to the guidelines: A prospective controlled study. J Dermatol Sci. (2017) 87:60–9. doi: 10.1016/j.jdermsci.2017.02.283 28314658

[36] Kolkhir P Giménez-Arnau AM Kulthanan K Peter J Metz M Maurer M. Urticaria. Nat Rev Dis Primers. (2022) 8:61. doi: 10.1038/s41572-022-00389-z 36109590

[37] Asady A Ruft J Ellrich A Hawro T Maurer M Altrichter S. Cholinergic urticaria patients of different age groups have distinct features. Clin Exp Allergy. (2017) 47:1609–14. doi: 10.1111/cea.13023 28873238

[38] Sibbald RG Cheema AS Lozinski A Tarlo S. Chronic urticaria. Evaluation of the role of physical, immunologic, and other contributory factors. Int J Dermatol. (1991) 30:381–6. doi: 10.1111/j.1365-4362.1991.tb03891.x 1894400

[39] Trevisonno J Balram B Netchiporouk E Ben-Shoshan M. Physical urticaria: Review on classification, triggers and management with special focus on prevalence including a meta-analysis. Postgrad Med. (2015) 127:565–70. doi: 10.1080/00325481.2015.1045817 25959894

[40] Kozel MM Mekkes JR Bossuyt PM Bos JD. Natural course of physical and chronic urticaria and angioedema in 220 patients. J Am Acad Dermatol. (2001) 45:387–91. doi: 10.1067/mjd.2001.116217 11511835

[41] Sánchez-Borges M Caballero-Fonseca F Capriles-Hulett A González-Aveledo L Maurer M. Factors linked to disease severity and time to remission in patients with chronic spontaneous urticaria. J Eur Acad Dermatol Venereol. (2017) 31:964–71. doi: 10.1111/jdv.14221 28299827

[42] Adachi J Aoki T Yamatodani A. Demonstration of sweat allergy in cholinergic urticaria. J Dermatol Sci. (1994) 7:142–9. doi: 10.1016/0923-1811(94)90088-4 7520273

[43] Takahagi S Tanaka T Ishii K Suzuki H Kameyoshi Y Shindo H. Sweat antigen induces histamine release from basophils of patients with cholinergic urticaria associated with atopic diathesis. Br J Dermatol. (2009) 160:426–8. doi: 10.1111/j.1365-2133.2008.08862.x 18811685

[44] Hiragun T Ishii K Hiragun M Suzuki H Kan T Mihara S. Fungal protein MGL_1304 in sweat is an allergen for atopic dermatitis patients. J Allergy Clin Immunol. (2013) 132:608–615.e4. doi: 10.1016/j.jaci.2013.03.047 23726042

[45] Oda Y Washio K Fukunaga A Imamura S Hatakeyama M Ogura K. Clinical utility of the basophil activation test in the diagnosis of sweat allergy. Allergol Int. (2020) 69:261–7. doi: 10.1016/j.alit.2019.09.003 31615718

[46] Kaplan AP Gray L Shaff RE Horakova Z Beaven MA. *In vivo* studies of mediator release in cold urticaria and cholinergic urticaria. J Allergy Clin Immunol. (1975) 55:394–402. doi: 10.1016/0091-6749(75)90078-0 48522

[47] Kaplan AP Garofalo J Sigler R Hauber T. Idiopathic cold urticaria: *in vitro* demonstration of histamine release upon challenge of skin biopsies. N Engl J Med. (1981) 305:1074–7. doi: 10.1056/NEJM198110293051808 6168912

[48] Houser DD Asbesman CE Ito K Wicher K. Cold urticaria. Immunologic Stud Am J Med. (1970) 49:23–33. doi: 10.1016/S0002-9343(70)80110-3 4194010

[49] Maurer M Schütz A Weller K Schoepke N Peveling-Oberhag A Staubach P. Omalizumab is effective in symptomatic dermographism-results of a randomized placebo-controlled trial. J Allergy Clin Immunol. (2017) 140:870–3. doi: 10.1016/j.jaci.2017.01.042 28389391

[50] Metz M Ohanyan T Church MK Maurer M. Omalizumab is an effective and rapidly acting therapy in difficult-to-treat chronic urticaria: a retrospective clinical analysis. J Dermatol Sci. (2014) 73:57–62. doi: 10.1016/j.jdermsci.2013.08.011 24060603

[51] Carballada F Nuñez R Martin-Lazaro J Juárez Y Castiñeira I Carballada F. Omalizumab treatment in 2 cases of refractory heat urticaria. J Investig Allergol Clin Immunol. (2013) 23:519–21. 24654322

[52] Carra S Dereure O Raison-Peyron N. A localized salt-dependent aquagenic urticaria successfully treated with omalizumab. Clin Exp Dermatol. (2022) 47:2339–41. doi: 10.1111/ced.15346 36131614

[53] Metz M Schütz A Weller K Gorczyza M Zimmer S Staubach P. Omalizumab is effective in cold urticaria-results of a randomized placebo-controlled trial. J Allergy Clin Immunol. (2017) 140:864–867.e5. doi: 10.1016/j.jaci.2017.01.043 28389393

[54] Church MK Kolkhir P Metz M Maurer M. The role and relevance of mast cells in urticaria. Immunol Rev. (2018) 282:232–47. doi: 10.1111/imr.12632 29431202

[55] Chang TW Chen C Lin CJ Metz M Church MK Maurer M. The potential pharmacologic mechanisms of omalizumab in patients with chronic spontaneous urticaria. J Allergy Clin Immunol. (2015) 135:337–42. doi: 10.1016/j.jaci.2014.04.036 24948369

[56] Nettis E Di Leo E Calogiuri G Foti C Macchia L. Successful treatment of four types of chronic urticaria with anti-IgE omalizumab in the same patient. Ann Allergy Asthma Immunol. (2019) 122:336–7. doi: 10.1016/j.anai.2018.12.002 30529714

